# Formation of substrate-based gold nanocage chains through dealloying with nitric acid

**DOI:** 10.3762/bjnano.6.140

**Published:** 2015-06-18

**Authors:** Ziren Yan, Ying Wu, Junwei Di

**Affiliations:** 1 The Key Lab of Health Chemistry and Molecular Diagnosis of Suzhou, College of Chemistry, Chemical Engineering and Material Science, Soochow University, Suzhou, Jiangsu 215123, PR China

**Keywords:** dealloying, gold nanocage chains, nitric acid, solid substrate

## Abstract

Metal nanocages have raised great interest because of their new properties and wide applications. Here, we report on the use of galvanic replacement reactions to synthesize substrate-supported Ag–Au nanocages from silver templates electrodeposited on transparent indium tin oxide (ITO) film coated glass. The residual Ag in the composition was dealloyed with 10% nitric acid. It was found that chains of Au nanocages were formed on the substrate surface during dealloying. When the concentration of HNO_3_ increased to 20%, the structures of nanocages were damaged and formed crescent or semi-circular shapes. The transfer process on the substrate surface was discussed.

## Introduction

Gold nanocages (Au NC) are a novel kind of nanostructure that possesses hollow interiors and porous shells [1–3]. Hollow metal nanostructures show unique physical and chemical characteristics with respect to their solid counterparts because of the high surface area, low density, and near-infrared localized surface plasmon resonance (LSPR). All these make Au NC an attractive material for various applications in optical [4–5] and electrochemical sensing [6], immunoassay [7], drug release [8], surface-enhanced Raman scattering (SERS), imaging [9], and catalysis [10–11].

Up to now, several methods, such as template-based methods, Kirkendall effect, Ostward ripening, and galvanic replacement, have been developed to synthesize hollow metal nanostructures [12–14]. Among them, the galvanic replacement method, which was first introduced by Sun and Xia [15], proved to be the most effective in producing gold nanocages. Because the AuCl_4_^−^/Au reduction potential is more positive than the Ag^+^/Ag potential (1.00 V and 0.80 V vs the standard hydrogen electrode, SHE, respectively), Ag is oxidized to Ag^+^ while AuCl_4_^−^ is reduced to Au. Thus, various Ag nanostructures have been employed as sacrificial templates for the generation of Au NCs. In general, four types of Ag nanostructures are used as sacrificial templates in replacement reactions: nanocubes and their truncated corners, octahedral nanostructures, and quasi-spherical nanoparticles [3]. During the replacement reaction, Au atoms are deposited epitaxially on the surface of the Ag template. They nucleate and grow into small islands, and eventually evolve into a shell around the silver particles. The thin shell formed in the early stage is incomplete, and the Ag dissolves to generate a hollow structure. This leads to the formation of Au NCs with hollow interiors and porous surfaces.

In some applications such as catalysis, sensors, and SERS, it is favorable for metal nanomaterials to be supported by a solid substrate. Although the fabrication of Au NCs from Ag templates dispersed in solution using galvanic replacement reactions has been carried out extensively, there are few reports for the preparation of Au NCs supported on a solid substrate through the galvanic replacement method [16–17]. Furthermore, in many cases, some residual Ag remains in the final Au NCs, which maybe limit their applications.

The dealloying or selective deletion of Ag from Ag–Au alloys with oxidative etchants such as nitric acid or perchloric acid has been widely used to synthesize nanoporous gold (NPG) [18–21]. Moreover, Xia et al. employed Fe(NO_3_)_3_ and H_2_O_2_ as dealloying reagent to selectively remove Ag atoms from Ag–Au alloy nanoboxes [4,22]. Here, we carried out galvanic replacement reactions on solid-supported Ag nanoparticle templates to form Ag–Au nanocages. Then, the residual Ag in the nanocages was selectively dissolved by chemical etchants. Interestingly, the Au NCs on indium tin oxide (ITO) film coated glass surface were mobilized to form chains in the treatment process with nitric acid. The discovery is used to prepare Au NC chains and advance the understanding of substrate-based dealloying reactions with nitric acid.

## Results and Discussion

### Synthesis of nanocages

**Figure 1:**
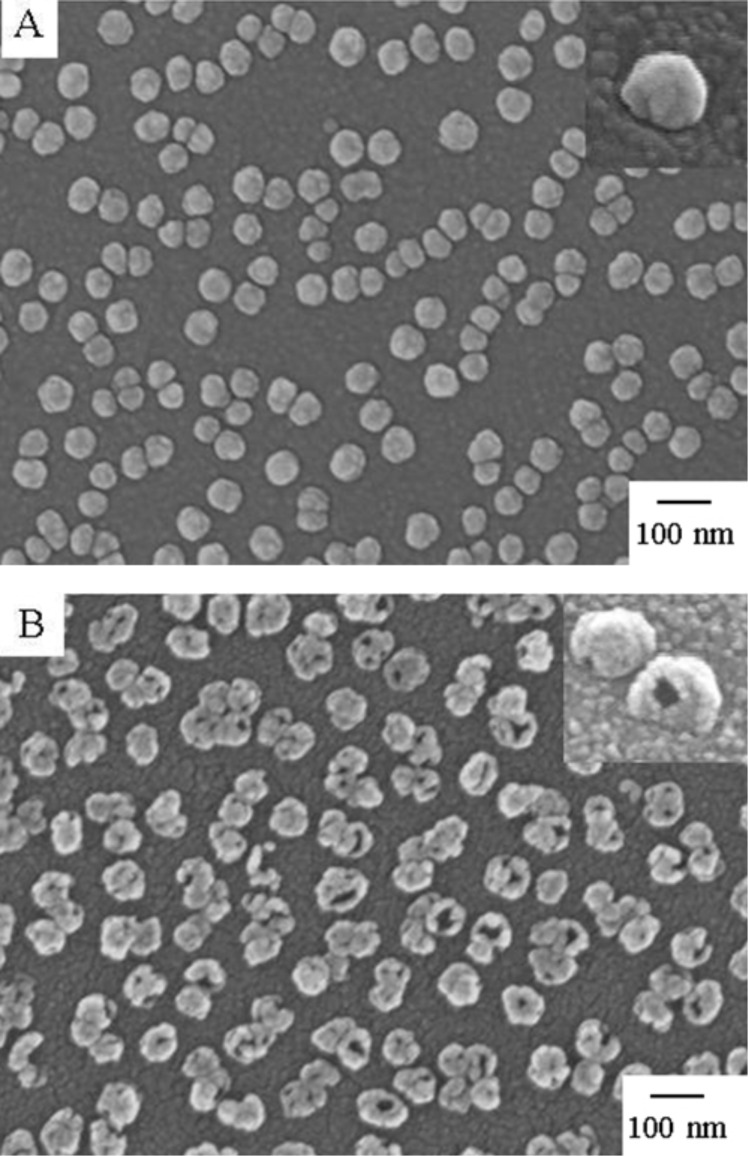
SEM images showing the morphology of AgNP templates before (A) and after (B) galvanic replacement using 0.1 mM HAuCl_4_ solution for 2 h with top-view. Insets: tilted-view with tilt angle of 45°.

In our previous reports [23–25], we have electro-deposited template silver nanoparticles (AgNPs) on ITO substrates and carried out the galvanic replacement reactions. Figure 1 shows top-view and tilted-view SEM images of unreacted AgNP templates and those exposed to aqueous 0.1 mM HAuCl_4_ solution for 2 h. Prior to the reaction the Ag templates show a morphology characteristic of semi-spheres in contact with a surface (Figure 1A). The average size of the AgNP templates was about 71 ± 9 nm. Also noteworthy, is that, unlike the routes based on chemical deposition process in solution, the templates are free of capping agents. When the Ag templates reacted with aqueous HAuCl_4_ solution, they underwent a morphological transformation to nanocages with hollowing of the interior and the appearance of openings at the structures (Figure 1B). The average size of the nanocages was increased to 83 ± 10 nm. This is attributed to the gold deposited on the surface of AgNP templates.

**Figure 2:**
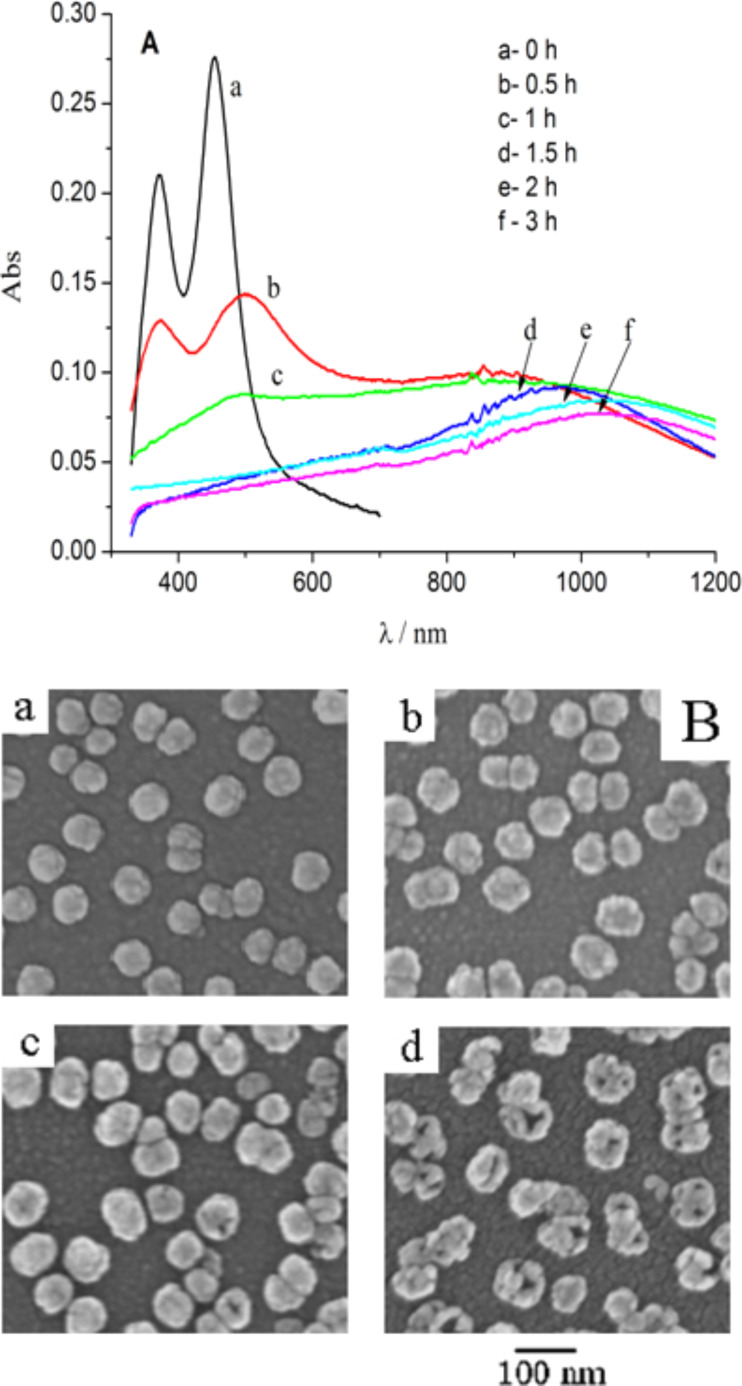
UV–vis–NIR absorbance spectra of ITO-supported AgNPs before and after reactions with 0.1 mM HAuCl_4_ solution for various times (A). SEM images of the AgNPs after reactions with 0.1 mM HAuCl_4_ solution for various times: a: 0 h, b: 0.5 h, c: 1 h, d: 2 h (B).

Since AgNPs, AuNPs and nanocages often exhibit different LSPR bands in the UV–vis–NIR wavelength region, the galvanic replacement reactions between the AgNPs and the aqueous HAuCl_4_ solution can be monitored using absorption spectroscopy. Figure 2A shows the UV–vis absorption spectra recorded from ITO-supported AgNPs after immersion in 0.1 mM HAuCl_4_ solution for various times. The spectra correspond to the excitation of dipolar and quadrupolar oscillations. The AgNPs exhibited typical LSPR peaks at 455 and 375 nm [26]. After incubation in 0.1 mM HAuCl_4_ solution for 0.5 h, the LSPR peaks of AgNPs decreased in intensity and the peak wavelength of the dipolar oscillation underwent a slight red shift. Meanwhile, a new LSPR peak located at ca. 900 nm appeared, which corresponds to the formation of Ag–Au nanocages. An alloying between the deposited Au and the underlying Ag may also occur because of the similar lattice constants [27]. After incubation for 1.5 h the peaks for the AgNPs almost disappeared (line d in Figure 2A). After longer incubation in HAuCl_4_ solution, the LSPR peak of the nanocages red-shifted continuously and decreased in intensity, owing to the larger void size, lower content of Ag, and the formation of holes on their surface. Furthermore, the LSPR band of the nanocages changed only little after more than 2 h of incubation, indicating the replacement reactions were nearly completed. The morphology transformation from AgNPs to Ag–Au NCs with HAuCl_4_ treatment time is also shown in Figure 2B. After 0.5 h of incubation, the nanoparticles exhibit a roughness similar to that of AgNPs. When reaction time increased to 1 h, small holes on the nanoparticles could be seen. As the reaction time reached 2 h, nanocage structures were formed.

**Figure 3:**
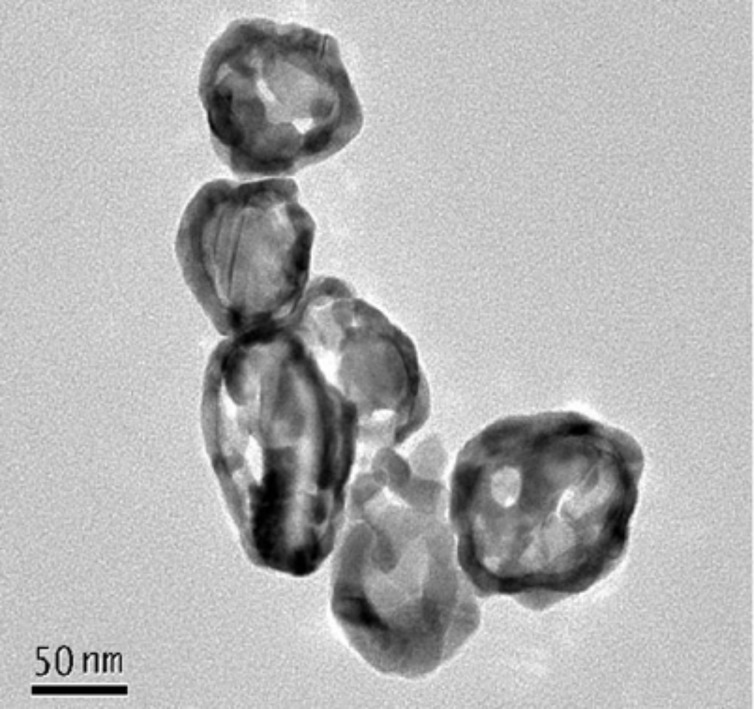
TEM image of Ag–Au NCs prepared by galvanic replacement reactions for 2 h and removed from the substrate surface.

In order to further demonstrate the formation of nanocages, the products of the replacement reactions removed from the substrate surface by sonication in ethanol solution. Figure 3 shows a TEM image of Ag–Au NCs released from ITO surface. It can be seen that hollow nanostructures were formed after galvanic replacement reactions. The thickness of the shell was measured to be approx. 8 nm, which is consistent with theoretical estimation of one-tenth of the radius of the corresponding silver template [28].

**Figure 4:**
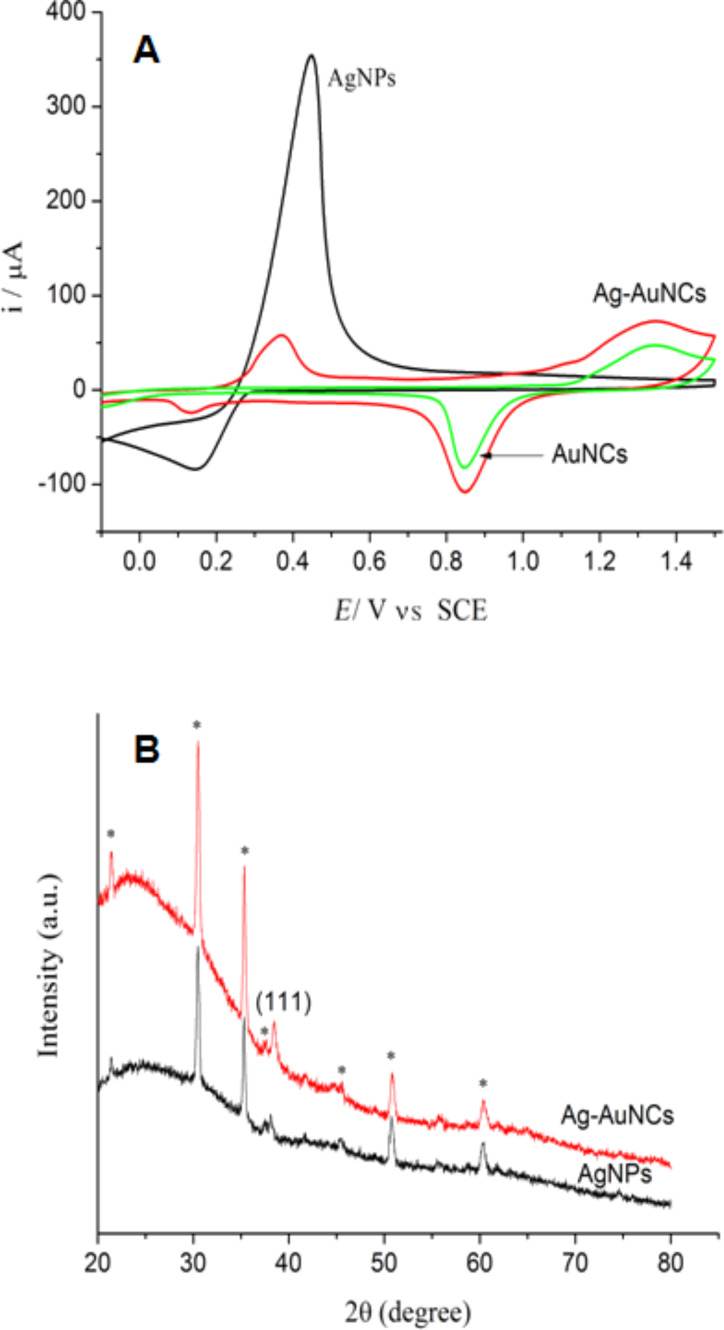
(A) Cyclic voltammograms of ITO-supported Ag NPs, Ag–Au NCs before and after (AuNCs) dealloying in 10% HNO_3_ solution for 10 h; (B) XRD patterns of ITO-supported Ag NPs before and after galvanic replacement reactions (* indicates the peaks of ITO).

In general, cubic Au NCs in solution were fabricated by galvanic replacement reactions at high temperature (boiling) under stirring [28–29]. However, the ITO-based nanocages were prepared by replacement reactions at 50 °C because high temperatures can release the nanomaterials from the ITO surface. Therefore, we used cyclic voltammetry to examine the composition of the metal nanomaterials. Figure 4A shows cyclic voltammograms of ITO-supported silver templates and nanocages in 0.05 M H_2_SO_4_ in the potential range from −0.1 to 1.5 V. It was observed that the redox peaks of Ag existed in the cyclic voltammograms of the nanomaterials (Ag–Au NCs) indicating that residual Ag remained in Ag–Au NCs. The result was consistent with a previous report that indicated the formation of most likely Au–Ag alloys at low temperature [29].

Figure 4B exhibits the XRD patterns of Ag NP templates and Ag–Au NCs. For pure AgNPs, only one small reflection was observed at 2θ = 38.2°, which corresponds to the (111) crystallographic planes of Ag. This suggested that (111) orientation is dominant in the Ag nanostructures. For Ag–Au NCs, the same reflection was observed at 2θ = 38.5°, corresponding to the (111) crystallographic plane. The similar peak position was expected after the replacement reaction because gold and silver have nearly the same lattice constant.

### Dealloying of Ag–Au NCs and formation of chains

**Figure 5:**
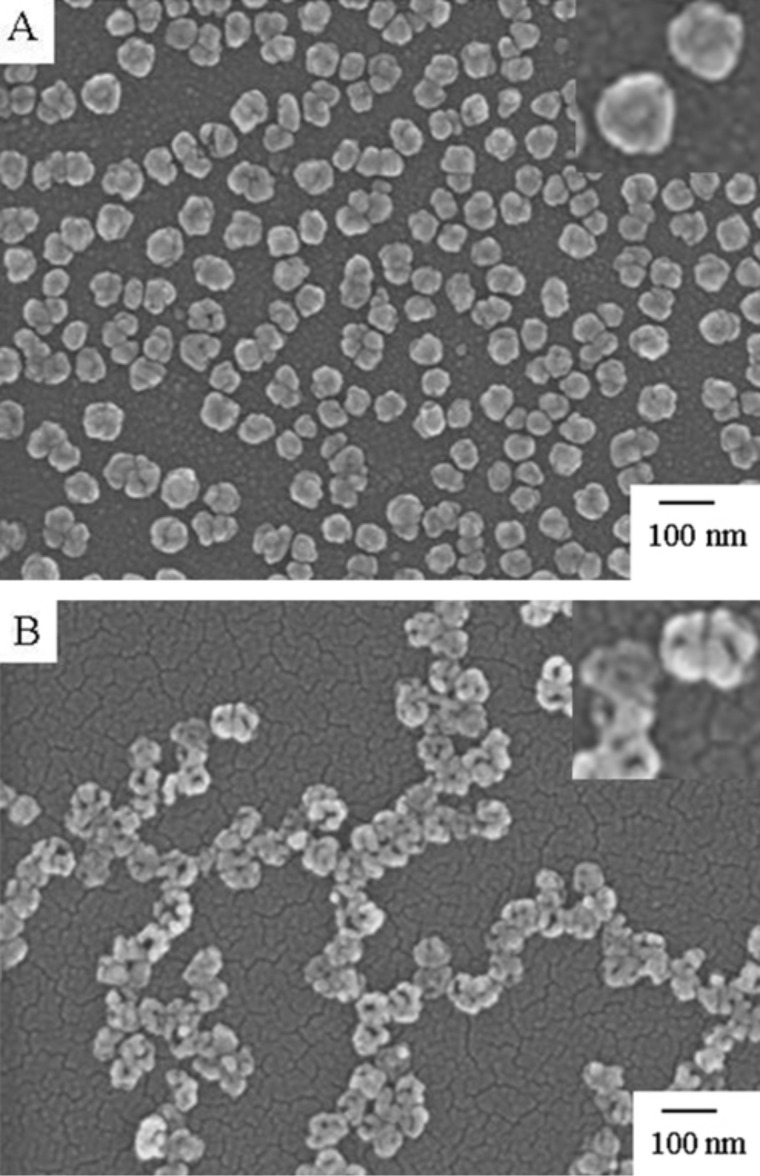
SEM images of Ag–Au NCs treated with 10% H_2_O_2_ (A) and 10% HNO_3_ (B) for 10 h at room temperature. Insets: the SEM images with higher magnification.

The pure Au nanomaterials (AuNCs) can be obtained by dealloying of Ag from Ag–Au NCs. Figure 5 shows the SEM images for Ag–Au NCs treated with 10% H_2_O_2_ and HNO_3_ for 10 h. When Ag–Au NCs were treated with 10 % H_2_O_2_ solution for 10 h, the holes in the walls of Ag–Au NCs became small or vanished but the particles were also distributed (Figure 5A). Furthermore, cyclic voltammetry indicated that some Ag was still residual in the nanoparticles, which is consistent with the previous report [4]. The chemical reaction involved in the dissolution of Ag at neutral conditions is the following:

[1]
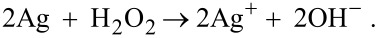


The deposition of byproducts, such as AgOH or Ag_2_O, on the particle surface might hinder the complete removal of Ag from the alloy and block up the hole of wall [30].

Nitric acid is generally used for dealloying Ag from Ag–Au alloys. The redox peaks of Ag disappeared in the cyclic voltammogram of the AuNCs (Figure 4A) indicating that the residual Ag was completely removed from the Ag–Au NCs and pure AuNCs were formed. This result was also confirmed by inductively coupled plasma atomic emission spectroscopy (ICP-AES) analysis of the dissolved metal nanoparticles. Figure 5B shows the SEM images of Ag-Au NCs treated with 10% HNO_3_ solution. It is obvious that the shape of nanocages changed little but the nanocages moved to form chains on the TIO substrate.

**Figure 6:**
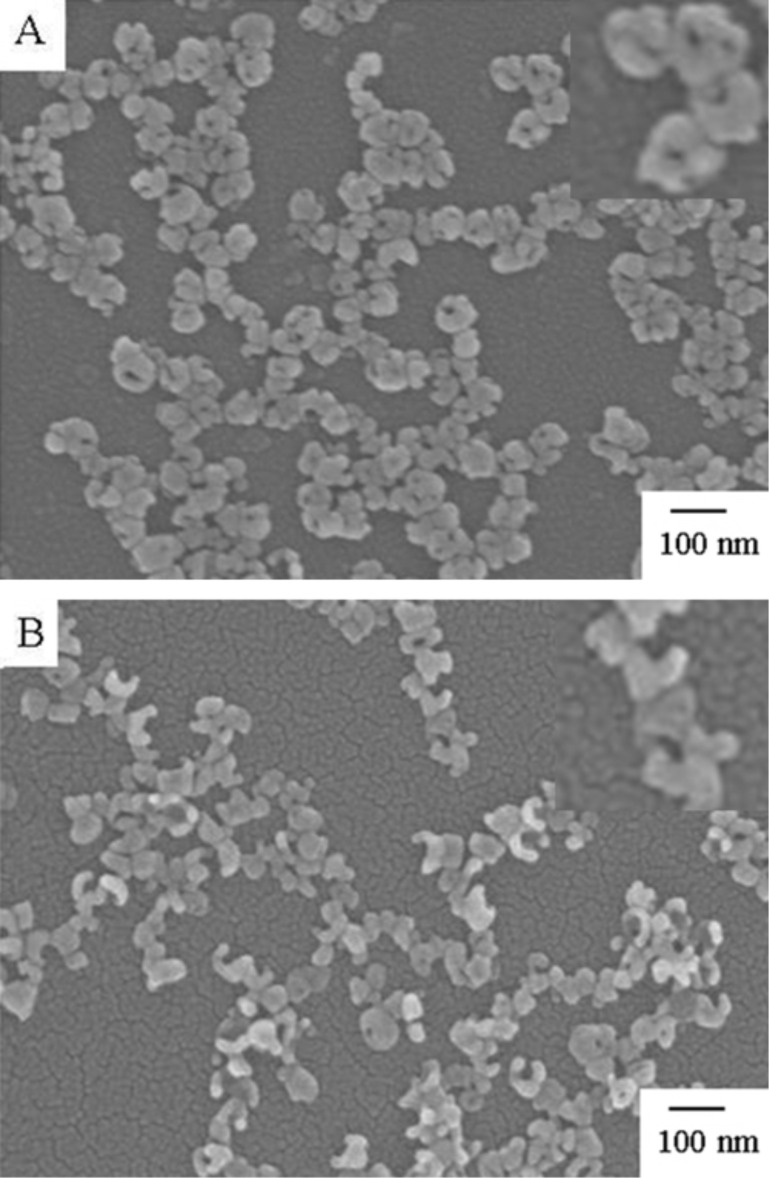
SEM images of Ag–Au NCs treated with 5% (A) and 20% HNO_3_ (B) for 10 h at room temperature. Inset: the SEM images with higher magnification.

It is reported that the concentration of HNO_3_ is an important parameter because it alters the chemical potential difference across the nanoporous/alloy interface and affects the morphology of Ag–Au alloy during dealloying. Figure 6 exhibits the SEM images of Ag–Au NCs treated with various concentration of HNO_3_. When the Ag–Au NCs were incubated in 5% HNO_3_ solutions for 10 h, the pores of particles were kept and the nanocages transferred to form chains on the ITO surface. This phenomenon was similar to that of the treatment with 10% HNO_3_ solution. As the concentration of HNO_3_ increased to 20%, the structures of nanocages were damaged to form crescent or semi-circular shapes (Figure 6B). When the concentration of HNO_3_ was over 30%, the particles were peeled off from the ITO substrate. This may be attributed to the rapid etching reaction in the highly concentrated nitric acid solution. This is different from the dealloying of Ag–Au alloy to form nanoporous gold, in which the concentration of HNO_3_ was rather high.

For a dilute HNO_3_ solution, the chemical reaction that may take place is given as follows [31]:

[2]
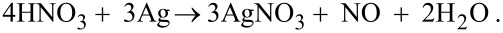


It is reported that the reaction is rather slow at HNO_3_ concentrations of less than 3.80 M (ca. 16%) [31]. A slow reaction could avoid the removal of the nanocages from the solid substrate. The gaseous product of the reaction may be the force to move the nanoparticles and the affinity between the gold nanoparticles may be the driving force of the chain formation. However, the details of the structure evolution mechanism are not clear. Therefore, the treatment with dilute HNO_3_ not only removes the residual Ag in the Ag–Au NCs but also initiates the migration of the nanocages.

## Conclusion

In conclusion, Ag–Au NCs were produced through substrate-based galvanic replacement reactions performed on electrochemically deposited silver templates. The residual Ag could be completely removed by incubation in dilute HNO_3_ solution. Moreover, by treating with 10% HNO_3_, the Au NCs could be mobilized to form chains on substrate. These synthesis and mechanism findings demonstrate the critical importance of HNO_3_ in dealloying Ag from the substrate-based Ag–Au NCs. It also points to potential opportunities in terms of engineering intricate nanostructures by using a combination of galvanic replacement reactions and dealloying procedures.

## Experimental

### Materials

The transparent ITO glass (1.1 mm, 100 Ω) was purchased from Suzhou NSG Electronics Co. Ltd. (Suzhou, China). Prior to use, it was divided into the strips (50 × 6 mm). All chemicals were of analytical grade and used as received without any further purification. Silver nitrate (AgNO_3_), hydrogen tetrachloroaurate (HAuCl_4_), nitric acid (HNO_3_), hydrogen peroxide (H_2_O_2_), and potassium nitrate (KNO_3_) were obtained from Sinopharm Chemical Reagent Co. Ltd. (Shanghai, China). The solutions were prepared using deionized water (>18 MΩ·cm).

### Silver template preparation

The Ag NP templates on ITO glass strips were prepared by electrodeposition in a similar manner as described in [23–25]. The electrochemical experiments were carried out with a RST 5200 electrochemical workstation (Suzhou Risetest Instrument Co., Ltd., China). A three-electrode system was used, including an ITO electrode as working electrode, a platinum wire as auxiliary electrode, and a saturated calomel electrode (SCE) as reference electrode. Prior to deposition, the glasses were cleaned using dilute NH_3_·H_2_O, ethanol, and water for 10 min sequentially in an ultrasonic bath. Then the ITO strip was put into 0.2 mM AgNO_3_ and 0.1 M KNO_3_ and the Ag nanoseeds were deposited on the ITO surface by a potentiostatic method applying a cathodic potential of −0.6 V for 3 s at 30 °C. Next, the AgNPs were grown by a square wave cyclic voltammetry in the range from −0.3 to approx. 0 V for 40 cycles with a frequency of 10 Hz, an amplitude of 25 mV and a potential step of 2 mV. Finally, the strips were rinsed with water and dried in a flow of nitrogen gas.

### Galvanic replacement reactions and dealloying

Ag–Au NCs were prepared by galvanic replacement reactions. Replacement reaction was carried out in a beaker with an aqueous 0.1 mM HAuCl_4_ solution (10 mL). The solution was kept in 50 °C water bath. Then the substrate-based silver templates were immersed for 2 h to form Ag–Au NCs. In order to realize dealloying, the strips were inserted in 10% HNO_3_ solution for 10 h in room temperature. Finally, they were rinsed with water and dried in atmosphere.

### Characterization

The surface morphology of nanoparticles was characterized with an S-4700 field-emission scanning electron microscopy (Hitachi, Japan). TEM images were obtained on a FEI Tecnai G2 20 transmission electron microscope. The nanoparticles on substrate were released into ethanol solution upon sonication. After that the sample was prepared by dropping the mixed solution on a copper grid. X-ray diffraction (XRD) analysis was performed by X’Pert-Pro MPD (Panalytical, Holland). The localized surface plasmon resonance (LSPR) spectra of nanoparticles were measured with a Shimadzu UV–vis–NIR 3600 Spectrophotometer against a bare ITO slide as the reference.
